# Supplementary material for: Lifestyle risk behavior and atherosclerotic cardiovascular risk: An analysis using the Korea National Health and Nutrition Examination Survey
Source: PLoS One. 2024 Aug 29;19(8):e0307677. doi: 10.1371/journal.pone.0307677 (PMC11361657; doi:10.1371/journal.pone.0307677)
Supplement: S1 Text — (DOCX) [file pone.0307677.s001.docx]

**Definitions for additional covariates**

Hypertension was defined as systolic blood pressure greater than 140 mmHg or diastolic blood pressure greater than 90 mmHg or the subject currently under treatment for hypertension.^1^ Diabetes was defined as fasting blood sugar greater than 126 (mg/dL) or HbA1c greater than 6.5% or the subject currently under treatment for diabetes.^2^ Educational years were defined as a categorical variable in the data with the criteria <7 years, 7-9 years, 10-12 years, and >12 years. The household income level was classified according to the quartiles of total household income by age groups. Occupation was categorized as unemployed, blue collar, and white collar with guidance from the Bureau of Labor Statistics job categories in the Standard Occupational Classification Manual.^3,4^ Marital status was defined as married if the subject is cohabiting with a spouse or if they are married.

**References**

1. Larson S, Cho MC, Tsioufis K, Yang E. 2018 Korean Society of Hypertension Guideline for the Management of Hypertension: A Comparison of American, European, and Korean Blood Pressure Guidelines. *Eur Heart J.* 2020;41(14):1384-1386.

2. Won JC, Lee JH, Kim JH, et al. Diabetes fact sheet in Korea, 2016: an appraisal of current status. *Diabetes Metab J.* 2018;42(5):415-424.

3. Chen A, Machiorlatti M, Krebs NM, Muscat JE. Socioeconomic differences in nicotine exposure and dependence in adult daily smokers. *BMC Public Health.* 2019;19(1):375.

4. Bureau of Labor Statistics - SOPC. 2010 SOC User Guide In: Standard Occupational Classification Policy Committee, https://www.bls.gov/soc. Accessed February 1, 2010.
