# Supplementary material for: Lifestyle risk behavior and atherosclerotic cardiovascular risk: An analysis using the Korea National Health and Nutrition Examination Survey
Source: PLoS One. 2024 Aug 29;19(8):e0307677. doi: 10.1371/journal.pone.0307677 (PMC11361657; doi:10.1371/journal.pone.0307677)
Supplement: S2 Text — (DOCX) [file pone.0307677.s002.docx]

**A detailed explanation of the Bayesian network mediation analysis**

A Bayesian network is a probabilistic graphical model that represents the relationships between variables in a dataset. In a Bayesian network, nodes represent variables and edges represent conditional dependencies between variables. A Markov Blanket is a set of variables that contains all of the variables that are direct causes, direct effects, or direct confounders of a given variable. The first procedure is building a Bayesian network that includes the predictor variable, the outcome variable, and all other variables that may influence the relationship between the predictor and outcome variables. In this procedure, the grow-shrink Markov Blanket algorithm enables to identify the Markov Blanket of the outcome variable. This will identify all of the variables that are directly or indirectly related to the outcome variable. After the comparison of the Markov Blankets of the outcome and mediator variables, we can determine whether the mediator variable lies on the causal pathway between the predictor and outcome variables. If the mediator variable is not in the Markov Blanket of the outcome variable but is in the Markov Blanket of the predictor variable, then the relationship between the predictor and outcome variables is mediated by the mediator variable.
