# Supplementary material for: Lifestyle risk behavior and atherosclerotic cardiovascular risk: An analysis using the Korea National Health and Nutrition Examination Survey
Source: PLoS One. 2024 Aug 29;19(8):e0307677. doi: 10.1371/journal.pone.0307677 (PMC11361657; doi:10.1371/journal.pone.0307677)
Supplement: Attachment — Submitted filename: comments plos one ASCVD.docx [file pone.0307677.s003.docx]

Dear editor,

Thank you for allowing me to review this manuscript.

The authors present a manuscript with valuable insights into the relationship between lifestyle risk behaviors and ASCVD risk in the Korean population, employing robust statistical methods and utilizing nationally representative data from KNHANES.

Here are my minor comments:

- In the discussion section; **the second paragraph lacks references**. “Lifestyle modifications, including smoking cessation and maintaining a high level of physical activity, are effective preventive measures that can reduce the CVD risk and maintain overall health. As described, many studies have reported that the CVD risk can be reduced by correcting multiple LRBs. However, it is clinically relevant to evaluate which of the LRBs precedes the effect modifier to reduce the CVD risk using limited preventive resources. To our best knowledge, only a few studies have 9 reported the causal relationship between several LRBs and CVD risk in a prospective cohort. Although this study used cross-sectional and retrospective data, the Bayesian network model was used to present which LRB component can probabilistically precede the other components.”
- I encourage the authors to provide a paragraph on the discussion section addressing how can healthcare professionals utilize this information to improve CVD risk assessment and prevention strategies.
